# Short-term forecasting of confirmed daily COVID-19 cases in the Southern African Development Community region

**DOI:** 10.4314/ahs.v22i4.60

**Published:** 2022-12

**Authors:** Claris Shoko, Caston Sigauke, Peter Njuho

**Affiliations:** 1 Department of Mathematics and Computer Sciences, Great Zimbabwe University. Private Bag 1235, Masvingo; 2 Department of Mathematical and Computational Sciences, University of Venda, Private Bag X5050, Thohoyandou, 0950, South Africa; 3 Department of Statistics, University of South Africa, South Africa

**Keywords:** Combined Forecasts, LQRA, PLAQR, OPERA, Quantile Regression Neural Networks, COVID-19

## Abstract

**Background:**

The coronavirus pandemic has resulted in complex challenges worldwide, and the Southern African Development Community (SADC) region has not been spared. The region has become the epicentre for coronavirus in the African continent. Combining forecasting techniques can help capture other attributes of the series, thus providing crucial information to address the problem.

**Objective:**

To formulate an effective model that timely predicts the spread of COVID-19 in the SADC region.

**Methods:**

Using the Quantile regression approaches; linear quantile regression averaging (LQRA), monotone composite quantile regression neural network (MCQRNN), partial additive quantile regression averaging (PAQRA), among others, we combine point forecasts from four candidate models namely, the ARIMA (p, d, q) model, TBATS, Generalized additive model (GAM) and a Gradient Boosting machine (GBM).

**Results:**

Among the single forecast models, the GAM provides the best model for predicting the spread of COVID-19 in the SADC region. However, it did not perform well in some periods. Combined forecasts models performed significantly better with the MCQRNN being the best (Theil's U statistic=0.000000278).

**Conclusion:**

The findings present an insightful approach in monitoring the spread of COVID-19 in the SADC region. The spread of COVID-19 can best be predicted using combined forecasts models, particularly the MCQRNN approach.

## Introduction

Coronaviruses, a large family of viruses, can cause illnesses that range from the common colds to much more severe diseases like SARS, Middle East respiratory syndrome, and COVID-19^1^. Signs of the COVID-19 disease may include fever, cough, shortness of breath and general breathing difficulties, organ failure, and even death. Some Chinese health authorities stated that coronavirus is likely to be transmitted from one person to another even before any symptoms (spread during the incubation period), making the epidemic difficult to prevent and control. This poses a severe threat to society as a whole. The Southern Africa region has been hit hardest by the COVID-19 pandemic in Africa, thus the epicentre of the coronavirus in the African continent^2^. Sixteen countries in the southern part of Africa constitute the SADC region namely Angola, Botswana, Eswatini, Comoros, Democratic Republic of Congo (DRC), Lesotho, Madagascar, Malawi, Mauritius, Mozambique, Namibia, Seychelles, South Africa, Tanzania, Zambia, and Zimbabwe. By February 2021 the SADC region had accounted for half of the reported cases in Africa. Of the five African countries accounting for close to 76% of new infections, three are members of the SADC, namely South Africa, Zambia, and Namibia^3^.

Forecasting is a part of statistical modelling widely used in various fields because of its benefits in decision making^4^. Forecasting is related to the formulation of models and methods that can be used to predict the future trend of uncertain situations. In most cases, one model is selected based on selection criteria, for example, the AICc, hypothesis testing and/or graphical inspection^5^. The model is considered to have the best performance accuracy forecast future values. However, this concept is only true if the model's premises are valid when applying it to the data. following Martinez et al.^6^: forecast models are based on the assumption that “the most reliable way to predict the future is to understand the present,” and, for this reason, these models do not say what will happen in the future, but say what can happen if the conditions observed in the present do not change. Thus, a bad model may casually predict the future better than a good model if the observed conditions in the present change radically in the future. A single technique cannot efficiently use a great deal of information due to the complexity of some time series. According to Bates and Granger,^7^ forecasting techniques have high accuracy when performing combination is achieved. Individual forecasting techniques based on different approaches capture distinctive characteristics of the series and allow for the combination to benefit from such characteristics^8^. A combined forecast allows for gathering available information, hence increasing the accuracy of the final forecast^9^.

ARIMA models have commonly been used in time series data analysis and forecasting and in predicting COVID-19 spread in particular^10^,^11^. Even though the ARIMA model is useful and powerful in time series analysis, sometimes it is difficult or rather cumbersome to identify the appropriate model for the data^12^. Recent results in machine learning show an improved performance of the final model not by choosing the model structure expected to predict the best but by creating a model whose results is the combination of the output of models having different formats. The various machine learning techniques applied are:

• **Generalized Additive Model (GAM):** These models assume that the mean of the response variable depends on an additive predictor through a link function. GAMs permit the response probability distribution to be any member of the exponential family of distributions.

• **Gradient Boosting Machine (GBM):** A decision tree model is chosen typically as a base model; however, an ensemble of such prediction model is chosen

• **Quantile Regression (QR) Models:** Standard linear regression focuses on finding a conditional mean function describing a linear relationship between the predictor and the independent variable(s). QR models look at different quantiles of the response defined by the conditional quantile function.

**i.** *Linear Quantile Regression (LQR) model:* The quantile regression model was introduced by Koenker and Bassett^39^, which models the relationship between predictor *X* and the conditional quantiles of *Y* given *X = x*. The linear quantile regression model complements the linear mean regression model if the error terms in the mean regression model are heteroscedastic.

**ii.** *Quantile Regression Neural Network (QRNN) Model:* the theoretical support for the use of quantile regression within an Artificial Neural Network to estimate potentially nonlinear quantile models.

**iii.** *Monotone Composite Quantile Regression Neural Network (MCQRNN) model:* estimates simultaneously multiple non-crossing quantile functions and allows optional monotonicity constraints

iv. Partial Additive Quantile Regression (PLAQR) averaging: Estimation, prediction, thresholding, transformation, and plotting for partial linear additive quantile regression.

• **Online Prediction by ExpeRt Aggregation (OPERA):** Considers a sequence of observations from a bounded time series to be predicted step by step. At each instant t, a finite set of experts, provides predictions x of the next observation in y.

Forecast combination methods exist and previous studies on forecasting show that combining forecasts generated from different models can considerably improve forecasting performance over single forecast models^13^. According to Zou and Yang5 combined forecasting improves accuracy performance. A fact confirmed by Adhikari and Agrawal14 is that combined forecasts lower forecast errors than individual models^14^. To the best of our knowledge. There is relatively no evidence of forecast combination in the context of COVID-19. This study introduces an efficient, flexible nonlinear quantile regression model, the monotone composite quantile regression neural network model to the modelling of the spread of COVID-19 in the SADC region.

In the next section we outline the methodology used in the study as well as formulation of the model. This is followed by the Results section where we explore the COVID-19 data for the SADC region and interpret results from fitted models. Lastly, we discuss and make conclusion based on the findings.

## Methods

### Data

**Figure 1:**
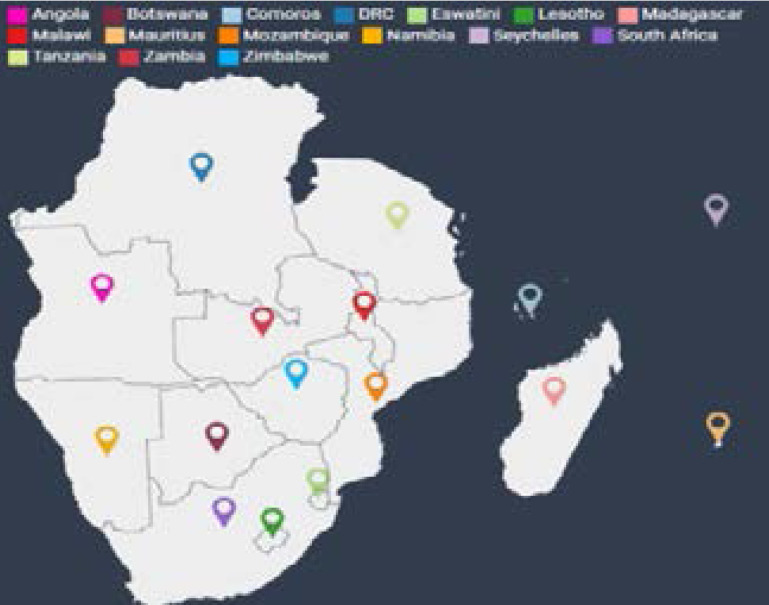
The spread of COVID-19 in the SADC region

In this study, we use an openly available daily number of confirmed cases of COVID-19 reported by Our World in Data (https://www.ourworldindata/coronavirus-source-data) from 7 March 2020 to 25 August 2021. We extract data from the daily confirmed cases for the SADC region. The SADC region is presented in Figure 1 below.

**Figure 2:**
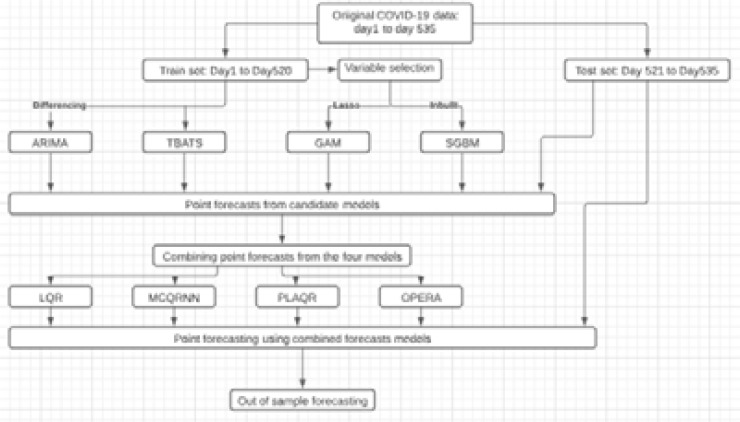
Schema for predicting the spread of COVID-19 in the SADC region.

Modelling and prediction of the spread of COVID-19 in the SADC region are done using the R packages: ‘forecast’ 15 for fitting the ARIMA and TBATS models, ‘gam’^16^ for fitting the generalized additive models, ‘gbm’^17^ for fitting the stochastic gradient boosting model, ‘qrnn’^18^ for fitting the linear quantile regression averaging and monotone composite quantile regression neural network model, ‘plaqr’^19^ and ‘opera’^20^. Figure 2 provides a schematic summary of the analysis procedure for predicting the spread of COVID-19 in the SADC region.

### Single Forecasting Methods

#### Non-Seasonal Autoregressive Integrated Moving Average (ARIMA) models

The growth of daily COVID-19 disease cases for the SADC region falls into the category of time series data, easily captured by an integrated model such as the ARIMA^21^. ARIMA models describe series that exhibit a trend that differencing can remove.

#### SARIMA Model

We have the general SARIMA model represented analytically as:


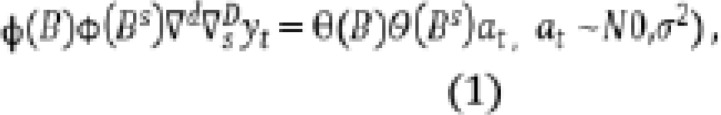


where *y_t_* represents the SADC confirmed daily cases on day 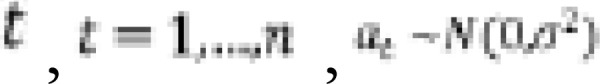 is the error term at time *t, s* is the seasonal length, *B* is a backshift operator (*Bz_t_ = z*_*t* - 1_). 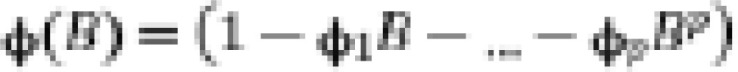 is the non-seasonal autoregressive (AR) operator, 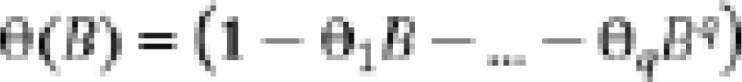 is the seasonal AR operator, 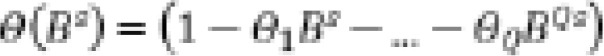 is the non-seasonal moving average (MA) operator, 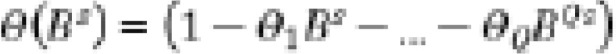 is the seasonal MA operator. ∇^*d*^ and 
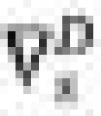
 are the non-seasonal and seasonal difference operators of order *d* and *D* respectively, where ∇^*d*^ = (1 − *B*)^*d*^ and 
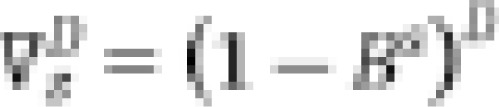
.

#### TBATS model

The TBATS model uses the Box-Cox transformation, exponential smoothing, trigonometric seasonality and ARMA errors^1^. It is generally used for forecasting time series with complex seasonal patterns. The components of the model are:

(i) The Box-Cox transformation



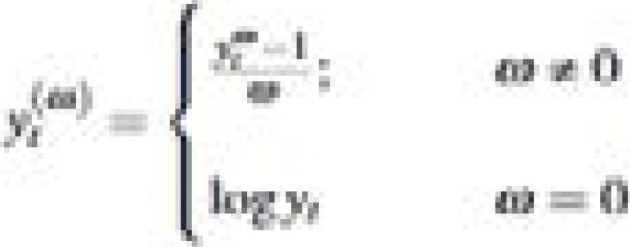



where *y_t_* is the confirmed daily cases on day *t, w* is the transformation parameter and denotes the natural logarithm.

(ii) Deterministic and stochastic trend



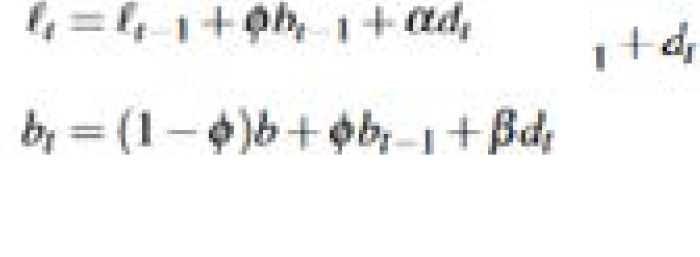



where *T* denotes the number of seasonal patterns 
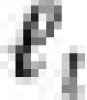
 is the local trend in period *t, b* represents the long-run trend, *b_t_* denotes the short-run trend in period 
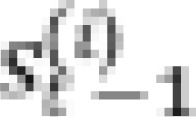
, represents *i*^th^ the seasonal component at time *t* − 1, *d_t_* denotes the ARMA (p, q) process and *α, β* and 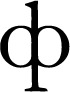 are smoothing parameters.

(iii) Trigonometric seasonality



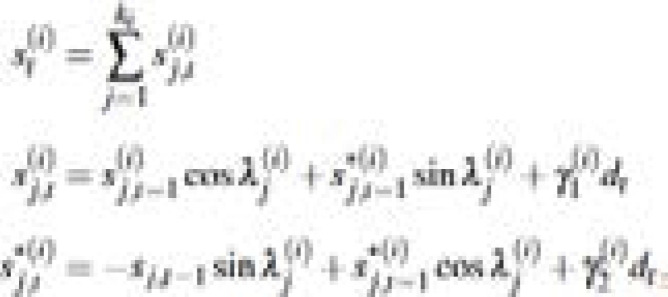



wher *y*^(*i*)^_1_ and *y*^(*i*)^_2_ are smoothing parameters and 
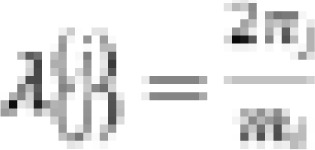
 with *m_i_* representing the period of the seasonal cycle.

(iv) ARMA errors



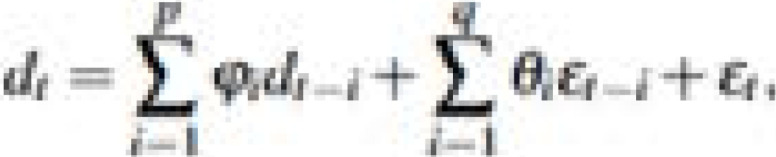



where *φ_i_, θ_i_* denote the autoregressive and moving average parameters, respectively and *ε_i_* is a white noise process.

The components (i) – (iv) put together give the TBATS model.

#### Generalized additive models

Let *y_t_* denotes the SADC confirmed daily cases on day *t, t* = 1,...,*n* with the corresponding covariates 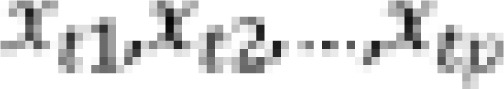, where *p* represent the number of variables. The generalized additive model is then written as:


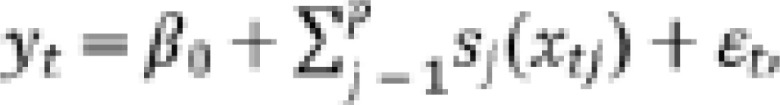


where *β*_0_ is a constant parameter, *s_j_* are smooth functions and *ε_t_* are independent and identically distributed (*i.i.d*) error terms. Equation (1) is estimated using penalized cubic splines^22^,^23^ given as:


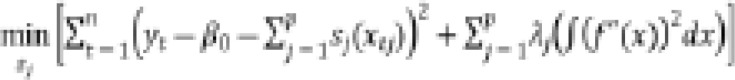



The penalty parameter controls the degree of smoothness 
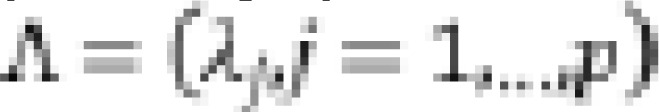
 which is optimized using the generalized cross-validation criterion (GCV)^23^. The smooth function, *b_i_*(*x*), is a sum of basis functions, , together with their regression coefficients *β_i_* and is given by 
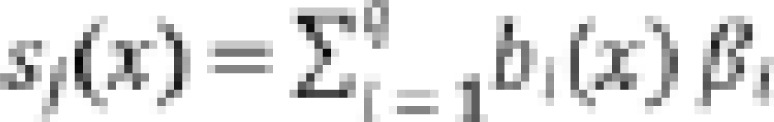
, where *q* denotes the basis dimension.

### Variable Selection

To reduce the problem of multicollinearity amongst the predictor variables we use the least absolute shrinkage and selection operator (Lasso). Lasso formulation is given as^24^,^25^:


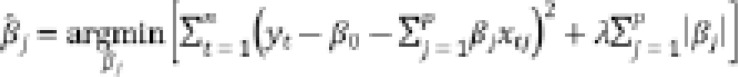


where λ is the shrinkage factor. The shrinkage factor, which lies between 0 and 1, is given by 
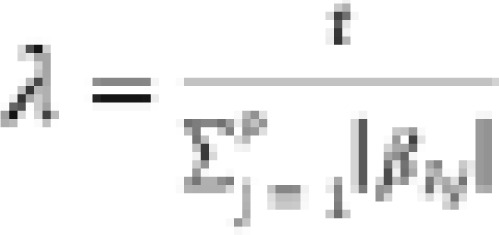
. See Tibshirani^24^ and Friedman et al.^25^ for a detailed discussion of Lasso.

### Stochastic Gradient Boosting Method (SGBM)

Gradient boosting (GB) is a machine learning technique that fits an additive model in a stage-wise way. The additive model can take the form given in Equation (8)^26^.


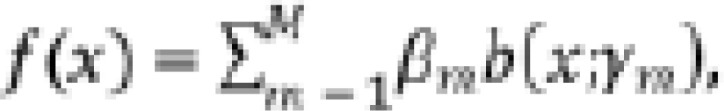


where 
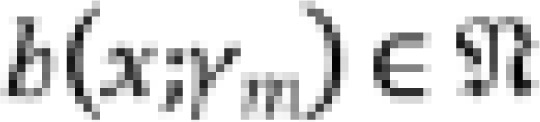
 are functions of *x* which are characterised by the expansion parameters *γ_m_, β_m_*. The parameters *β_m_* and *γ_m_* are fitted in a stage-wise way, a process which slows down over-fitting^26^. Stochastic gradient boosting (SGB) is an extension of GB in which a random sample of the training data set is taken without replacement. See Friedman for a detailed discussion of the gradient boosting method^27^.

### Combining Forecasts

Combining forecasts was first developed by Bates and Granger^7^, who argued that combined forecasts improve forecast over the single model forecast. Suppose the point forecasts from the ARIMA, TBATS, GAM, and SGBM models are combined so that we have a vector


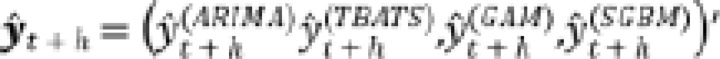



Then, the combined forecasts for this vector are obtained using the LQRA, MCQRNN, PLAQR, and OPERA approaches. The accuracy of the performance of these models is checked by comparing their respective RMSE, MAPE and Theil's U statistics.

### Quantile Regression Averaging (QRA)

**Figure 3:**
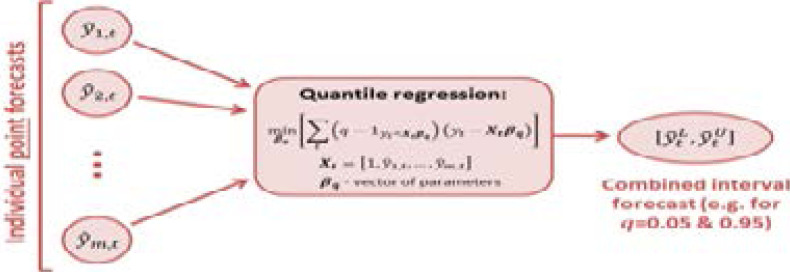
Quantile regression averaging.

In the standard QR setting, individual point forecasts are used as independent variables and the corresponding target variable as the dependent variable^28^. The relationship between the predictor and the independent variable(s) is not described with a single slope parameter just like in linear regression models, but a set of parameters *β_τ_* dependent on the quantile *τ* must be estimated. We define the *τ^th^* regression quantile ( ) as any solution, to the quantile regression minimization problem^29^:


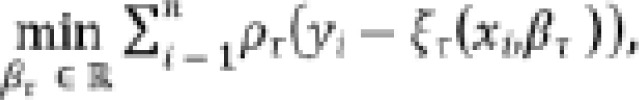


where 
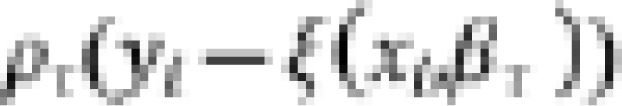
 is a function of *τ* and 
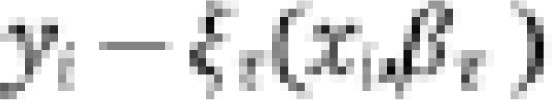
. This kind of loss function is most often called check or pinball loss function and is defined as follows:


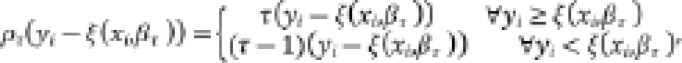


where 
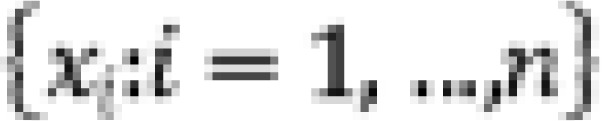
 denotes a sequence of explanatory variable and 
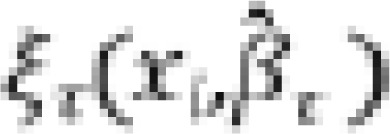
 is formulated as a linear function of parameters. The LQRA model is given by


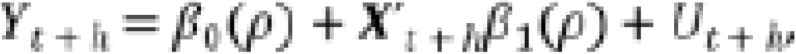


where 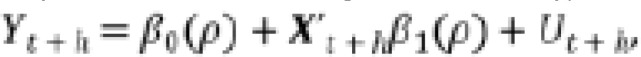 and *ρ* is the probability mass of interest. *X_t + h_* is a vector of covariates for the *t + h* forecasted value from the fitted ARIMA model, TBATS model, GAM, and SGBM, i.e., multivariate quantile regression model. The unknown parameter vectors appearing in the above equation can be solved from the following optimization problem:


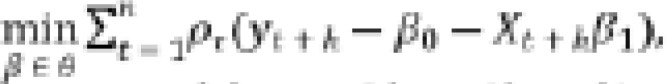


where 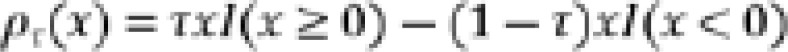 is the check function. Figure 3 presents a schematic expression of the quantile regression average.

Figure 3 shows the link between the individual point forecasts through the quantile regression to the combined interval forecast.

### Monotone Composite Quantile Regression Neural Network (MCQRNN)

MCQRNN is a novel form of quantile regression that can be used to simultaneously estimate multiple non-crossing. It combines elements drawn from the QRNN model^30^,^31^, the monotone multilayer perception (MMPP)^32^, the composite QRNN^33^, the expectable regression network^34^ and the generalized additive neural network^35^. Cannon18 gives an elaborate explanation on the formulation of the MCQRNN.

### Combining prediction intervals

Robust prediction intervals are known to be produced from combining prediction limits from various models^36^,^37^,^38^. We use the simple average and median methods for combining the prediction limits. The simple average method uses the arithmetic means of the prediction limits from the forecasting models. Thus, expressed as


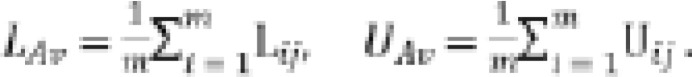


The median method is known to be less sensitive to outliers and is given in Equation 16.


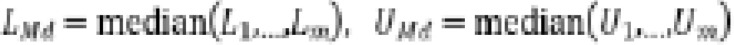


For each of the models, 
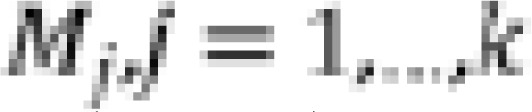
, we compute the prediction interval widths (PIWs), which we denote by 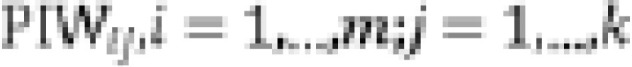 and calculate as


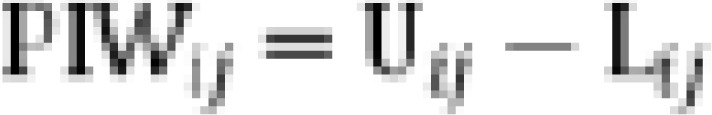


where *U_ij_* and *L_ij_* are the upper and lower limits of the prediction interval, respectively. Various indices are used to evaluate the reliability of prediction intervals (PIs). In this study we use the prediction interval normalised average width (PINAW). We express the PINAW, an index that check if the required value is covered by the prediction interval as


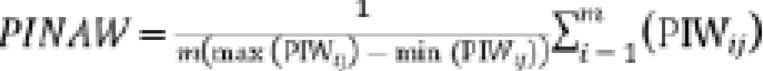


Using PINAW we compare different models and then determine the one that possesses the smallest percentage value.

### Empirical Results

#### Exploratory data analysis

We use an openly available daily number of confirmed cases of COVID- 19 reported by Our World in Data (www.ourworldindata/coronavirus-source-data) from 7 March 2020 to 25 August 2021. The number of daily reported cases for the SADC region ranged from 0 to 32 321. From 7 March 2020 to 3 August 2021, the average number of reported cases was 6 581 per day.

**Figure 4:**
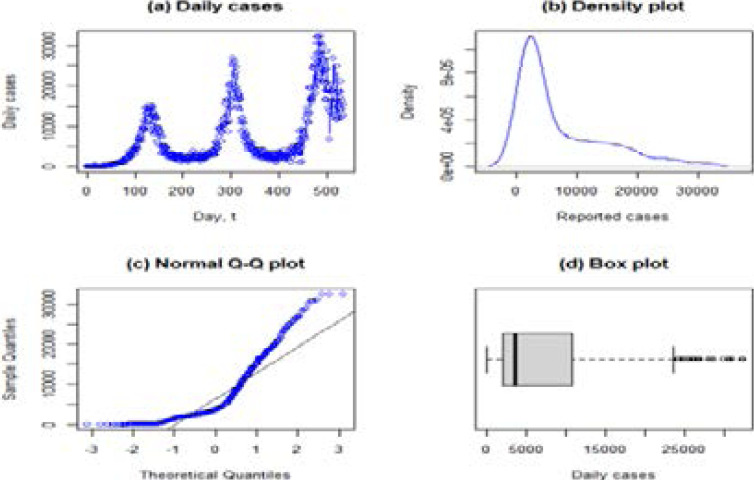
Normality checks for the daily COVID-19 series

We further perform a univariate data analysis for the reported daily COVID-19 cases by plotting the time series data and the density plot, normal Q-Q plot and the Box plot as shown in Figure 4. The plots check for the normality assumption in the time series data.

Figure 4(a) presents the time series trend for the daily COVID-19 cases in three phases. The first phase has the lowest peak and the peaks increase with time, with the third phase having the highest peak. Figure 4(b) presents the density plot, which shows that the series is positively skewed, thus, not normally distributed. Figure 4(c) is the Q-Q plot. The deviations from the diagonal line in the normal Q-Q plot imply that the data extend farther out than expected under normality. A correlation matrix showed some highly correlated variables (see the correlation matrix given in the supplementary material). We use Lasso (discussed in Section 2.2.4) to reduce the multicollinearity problem in variable selection.

#### Predictive modelling for the reported daily COVID-19 cases in the SADC region

The series is relatively long and can be divided into train and test sets. The training set constitutes the first 520 observations and 521 to 535 represent the test set. In the next section, we fit the ARIMA model, TBATS, Generalized Additive Model and Stochastic Gradient Boosting for the training set and use the fitted models to check if they fit the test set well.

### Time series ARIMA model

**Figure 5:**
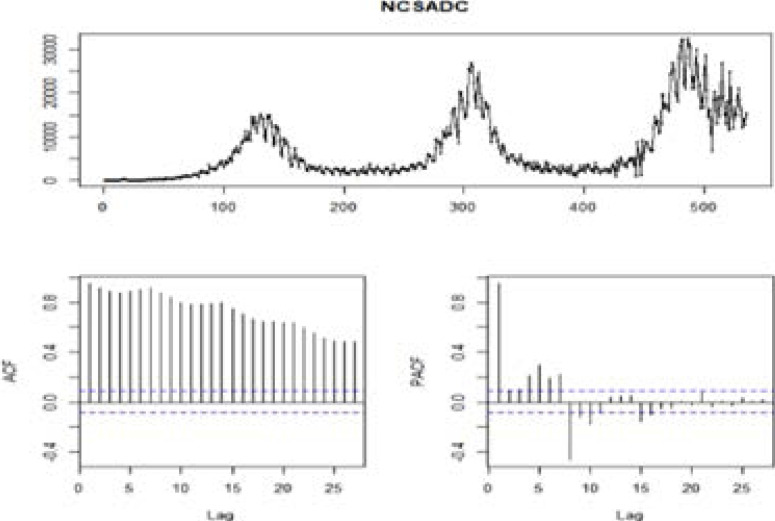
Display of the time series for the daily COVID-19 cases in the SADC region.

We start by testing for the stationarity of the original time series data and that of the differenced time series data. This is done using the augmented Dickey-Fuller (ADF) test and the Kwiatkowski-Phillips-Schmidt-Shin (KPSS) test at a 5% level of significance. ADF test: the null hypothesis is that the data are non-stationary and non-seasonal. KPSS test: the null hypothesis is that the data are stationary and non-seasonal. A plot of the residuals autocorrelation function (ACF) is also used to investigate the stationarity of the original time series. Figure 5 present the results.

The ACF plot shows that all autocorrelations are outside the threshold limit. This indicates that the original series is not white noise. A Scatter plot of residuals shows that yt is correlated to yt-1.

Further diagnostic of residuals using the Box-Pierce test and the Box-Ljung test return small p-values <2.2e-16, suggesting that the original series is not white noise. The ADF (-2.2052, p-value=0.4915) and the KPSS tests (1.9668, p-value=0.01) show that the original time series is not stationary. After the first difference of the data, both the ADF and KPSS test show that the differenced series is stationary in its mean and variance at 5% level, p-values= 0.01 and 0.1 respectively. Therefore, we adopt d = 1 for ARIMA (p, d, q) model.

The ACF and PACF charts for the differenced time series, though not shown, were used to help select the candidate ARIMA models by observing the spikes in the ACF and PACF. The spikes in the PACF plot suggest an AR^7^ and ACF suggest a MA^7^. Thus, the initial candidate model takes the form of ARIMA^7^,^1^,^7^. We consider several ARIMA models, including the auto-selected ARIMA model and assess the accuracy of their performance, based on the AICc. The ARIMA^14^,^1^,^8^ with the lowest AICc (AICc=9102.03) compared to all the other ARIMA models is considered the best ARIMA model for predicting the spread of COVID-19 in the SADC region. At 5% significance level, the Box-Ljung test ( -squared = 8.9064, df = 20, p-value = 0.984) shows that the residuals for the fitted ARIMA^14^,^1^,^8^ model are stationary.

### TBATs

The best TBATS model for the confirmed daily COVID cases for the SADC region is a BATS (1, {3,2}, 0.886, -) where Box-Cox transformation is 1 (doing nothing).

### Generalized additive model

Before fitting the GAM and the GBM we created some covariates where


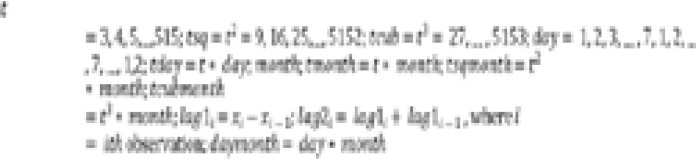



The model for the GAM and GBM is:


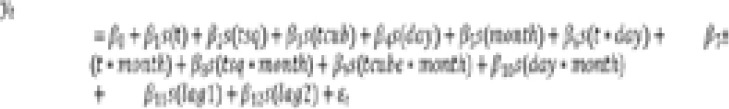



Before fitting using Lasso shrinkage approach discussed in Section 2.2.4, we fitted the GAM because it does not have an inbuilt mechanism for variable selection. Table 1 presents the results for the fitted model (18).

**Table 1:** Selection of variables for the GAM via the Lasso approach

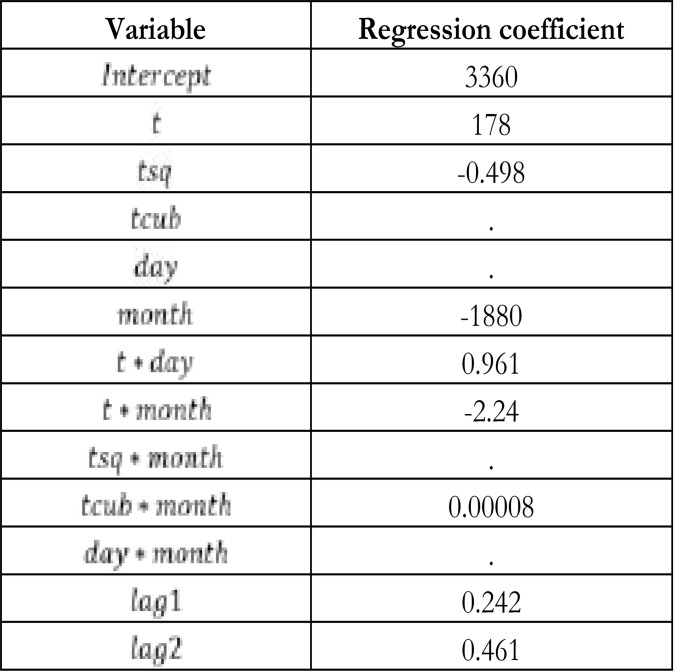

Table 1

**Figure 6:**
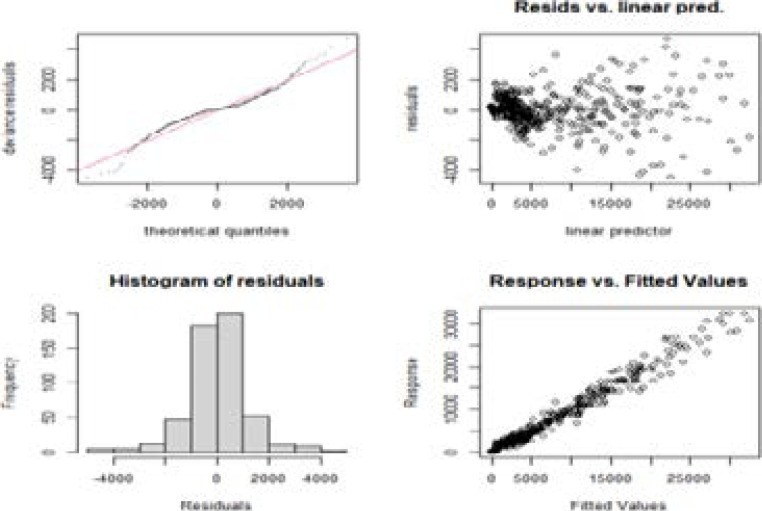
Normality checks for the fitted Generalized Additive Model

The results in Table 1 show that the variables t * cub, day, tsq * month and day * month do not contribute significantly to the GAM and hence exclude them in the building of the GAM model. Figure 6 presents plots that check for the normality assumptions in the fitting of GAM.

The graphs in Figure 6 theoretical quantile plot's tails. This suggests a rather heavy tail distribution, different from a normal distribution. In addition, the plot of residuals shows strong heteroscedasticity.

### Stochastic Gradient boosting method (SGBM)

Unlike the GAM, the SGBM has an inbuilt mechanism for selecting variables. Table 2 shows the influence of the variables on the fitted SGBM. The variables are ranked from the most influential to those that do not influence the fitted model.

**Table 2:** Selection of variables for the SGBM

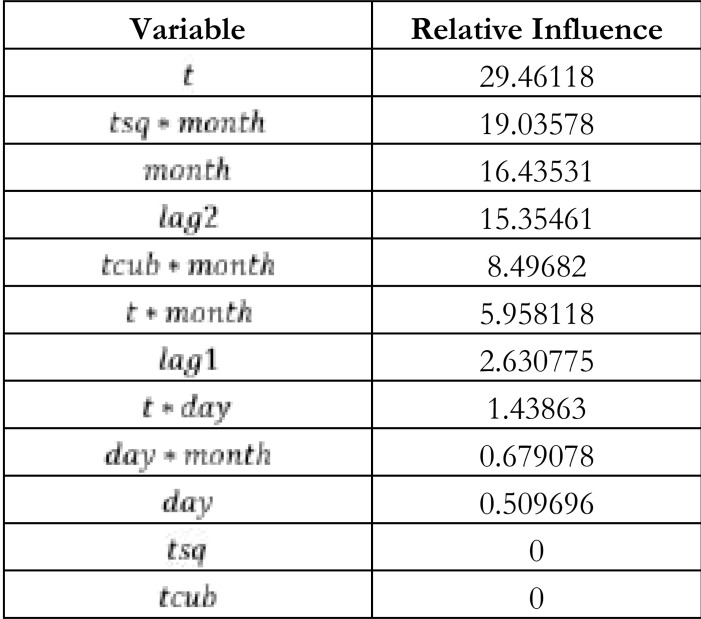

Table 2 results show that the variables , and have a zero influence on the fitted model.

We display the predicting outputs for all the fitted models namely the ARIMA (14,1,8), TBATS, GAM and Stochastic gradient boosting model (SGBM) in Table 3. Included in the table are the forecast performance measures namely the RMSE, MAE, MAPE and Theil's U results.

**Table 3:** Forecast performance measures for the single forecast models

Model	RMSE	MAE	MAPE	Theil's U
SGBM	1906.75	1658.871	10.54381	0.3356
GAM	1459.164	1158.378	7.81968	0.2387
TBATS	2610.837	2091.848	14.19489	0.3880
ARIMA (14,1,8)	2602.141	2372.979	16.2012	0.4747

Among the four models, the GAM performed best in the prospective forecasting of daily COVID-19 cases over the following 15 days, with the smallest values of RMSE (1459.164), MAE (1158.378) and MAPE (7.81968). The SGBM showed better goodness of fit than the ARIMA^14^,^1^,^8^ and TBATS models. For the forecast accuracy, the ARIMA^14^,^1^,^8^ showed a greater RMSE (2602.141) than the GBM (1906.75), as well as a greater MAE (2372.979 vs. 1658.871) and MAPE (16.2012 vs. 10.54381).

Table 4 presents the forecasting results from the fitted ARIMA^14^,^1^,^8^ , TBATS, GAM and SGBM models.

**Table 4:** Forecasting from the ARIMA (14,1,8), TBATS, GAM and SGBM

		*ŷ* (*ARIMA*) *t + h*	*ŷ* (*TBATS*) *t + h*	*ŷ* (*SGBM*) *t + h*	*ŷ* (*GAM*) *t + h*
**Forecasts(*h*)**	1	15011.18	18366.55	17254.98	14445.34
	2	24344.25	20869.12	24742.36	21662.12
	3	20593.48	20632.58	23556.69	18847.04
	4	18784.38	18209.17	17652.61	13026.52
	5	15970.79	15357.42	17374.73	11163.43
	6	18708.81	14073.74	17915.55	14706.16
	7	15575.50	15146.23	20528.35	16241.85
	8	15556.11	17594.13	21731.04	17235.09
	9	23111.45	19438.31	24227.89	18920.46
	10	19832.68	19204.77	17110.47	14820.07
	11	18226.37	17019.44	17652.61	13729.31
	12	14903.75	14480.82	17374.73	10198.84
	13	17213.17	13430.18	18457.68	12865.33
	14	15787.95	14563.86	16915.97	13778.62
	15	15048.45	16932.37	18629.08	15226.46

The results in Table 4 show that the GAM, the model that performs the best compared to the rest, predicts the lowest number of COVID-19 cases than all the other models.

**Figure 7:**
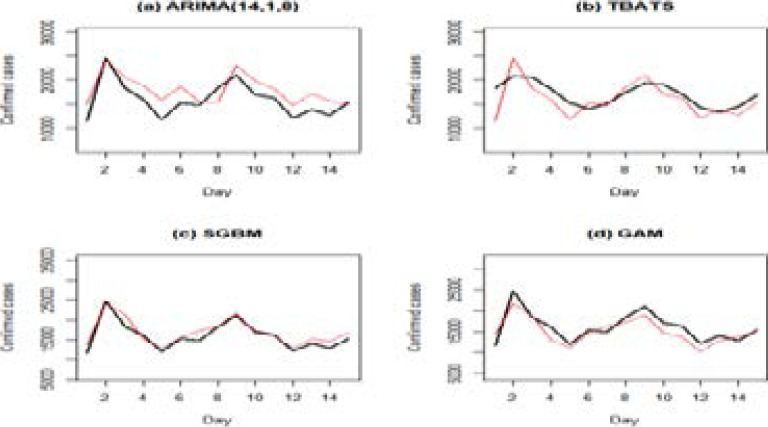
Plot of the 15-day forecast from the training set (predicted values) and the test set, the black line represents observed/actual values of the test set

Figure 7 (a-d) displays comparison plots of the 15-days forecast from the training set and the test set (observed series) of the fitted models. The black line represents observed/actual values of the test set. Figure 7(a) presents the forecast from the training set for the ARIMA^14^,^1^,^8^ model and the test set, (the observed series). Figure 7(b) presents the prediction from the TBATS model. Figure 7(c) presents the prediction from the SGBM and Figure 7(d) represents the prediction from the GAM.

Figure 7(a-d) plots show that prediction from the GAM gives a better fit of the test set (observed set) followed by the SGBM, the ARIMA^14^,^1^,^8^ and lastly the TBATS model. Although the GAM is the best of the four fitted models, it does not perform well in certain periods while other models perform better in other periods. Therefore, we suggest combining forecasts from the ARIMA^14^,^1^,^8^ model, TBATS, GAM, and the SGBM to improve forecasts over individual models.

### Combining forecasts

Let the vector of forecasters from the ARIMA (14,1,8) model, TBATS model, GAM and SGBM be


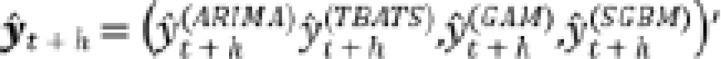



We combine the forecasters using four different methods namely the Linear Quantile Regression (LQR) model, Monotone Composite Quantile Regression Neural Network (MCQRNN) model, Partial Linear Additive Quantile Regression (PLAQR) averaging, and Opera. For the first three (LQR, MCQRNN, and PLAQR) the value of the conditional quantile, gives better forecasts. Table 5 presents results of the comparison of the RMSE, MAE, MAPE, and Theil's U statistic for the fitted models, used to check the accuracy of the performance of the combined forecasts.

**Table 5:** Forecast performance measures for the combined forecast models

Forecast combination model	RMSE	MAE	MAPE	Theil's U
LQR	1196.701	703.655	4.014866	0.1896
MCQRNN	0.001323	0.001049		
PLAQR	351.5644	111.6311	0.72325	0.0611
OPERA	1244.245	936.4002	6.264443	0.1970

Results in Table 5 indicate that the RMSE and MAPE for combination forecast models (LQR, MCQRNN, PAQR, OPERA) are lower than the RMSE and MAPE for the single forecast models (ARIMA, TBATS, GAM, GBM). Thus, forecast combinations improve the accuracy over the single forecast models for the daily COVID-19 cases for the SADC region. The MCQRNN has the lowest RMSE=380.931 and MAPE=0.808865, compared to the other models. Theil's U statistic for the MCQRNN model is close to 0 suggesting a perfect fit for the forecast.

**Figure 8:**
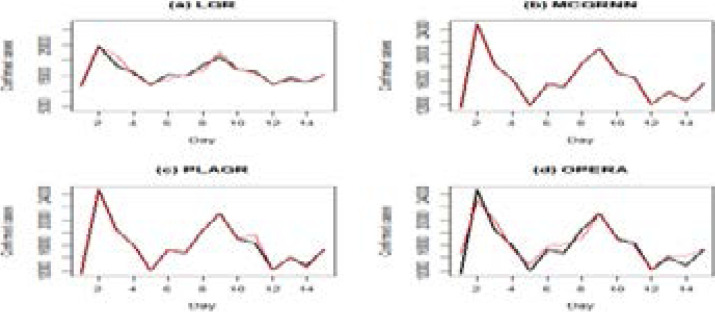
The comparison of forecasts from combined forecast models where the red line represents the predicted values from the training set and the black line represents observed/actual values of the test set

Figure 8 shows a further comparison of the performance of the combination forecast models. We visualize how good forecasts from the training data set fits the testing set. The testing set rep resents the original series, which as explained earlier constitutes the last 15 of the observed data.

Figure 8 results reveal that the MCQRNN model fits the observed series (test set) well. The plot of the MCQRNN shown in black is closer to the plot of the test set. Although the PLAQR also gives a better fit, it does not provide a good prediction for 10–12 days. Thus, the MCQRNN model outperforms all the other combination models hence, the preferable model.

### Out of sample forecasts

We use the developed models for out-of-sample prediction of the confirmed daily cases of COVID-19. Table 6 presents the instances of the predicted cases for the next 14 days, ranging from 26-08-2021 to 08-09-2021.

**Table 6:** Predicted cases for the next 15 days (26-08-2021 to 08-09-2021)

*t + h*	*ŷ* (*ARIMA*) *t + h*	*ŷ* (*TBATS*) *t + h*	*ŷ* (*SGBM*) *t + h*	*ŷ* (*GAM*) *t + h*	L.95_med	*ŷ* (*combined*) *t + h*	U.95_med
536	19600.88	17649.11	24652.49	22166	14218.63	**22194.46**	26630.79
537	17038.81	17263.71	18486.34	15722	13427.25	**16764.91**	21100.17
538	18070.16	15091.17	16263.04	13946	10971	**15600.43**	19211.35
539	12547.15	12715.74	13962.07	12528	8436.59	**10831.03**	16994.88
540	13526.01	11844.08	15577.29	13104	7452.54	**12911.73**	16235.62
541	15958.47	13039.32	16688.39	15052	8495.5	**14541.09**	17583.14
542	15992.99	15326.47	16723.41	18052	10509.49	**13252.8**	20143.45
543	19571.18	16944.35	20527.88	21879	11727.81	**17673.14**	22160.89
544	16607.93	16664.65	17424.34	17328	11034.46	**14654.66**	22294.83
545	18057.55	14690.51	16490.99	16624	8746.95	**14684.7**	20634.07
546	13177.78	12477.85	14171.26	14983	6332.65	**10319.51**	18623.05
547	13531.66	11632.73	14617.19	15061	5334.77	**10981.04**	17930.69
548	15887.62	12719.35	14933.97	16719	6240.52	**11805.66**	19198.17
549	15984.92	14862.81	16581.5	17837	8109.93	**13189.7**	21615.69

Results in Table 6 indicate that the number of new confirmed COVID-19 cases fluctuates between 1297 and 23000 for the next 14 days, that is from 26 August 2021 to 8 September 2021. A downward trend in the number of confirmed cases is occurring.

### Evaluation of Prediction Intervals

**Figure 9:**
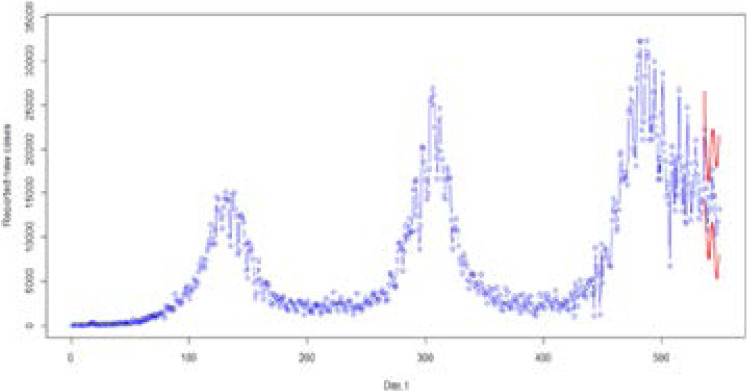
Plot of the confirmed cases including forecasted cases for the period 26-08-2021 to 08-09-2021 with the 95% prediction interval. The prediction intervals are from the median combination method for combining prediction limits

We also assess the sharpness of the predictive distributions by calculating the prediction intervals normalized average width (PINAW) using the methods discussed in Section 2.3.2, i.e., from simple average and median. All the prediction intervals are at the 95% level. The computed PINAWs for the models are 2.5795 and 1.8285 for the simple average and median, respectively. The median has a narrower prediction interval than the average. Figure 9 presents plot of the confirmed cases including forecasted cases for the period 26-08-2021 to 08-09-2021 with the 95% prediction interval. The prediction intervals are from the median combination method for combining prediction limits.

## Discussion

Improvement of time series forecasting accuracy through combining forecasts from multiple time series candidate models is an important and dynamic area of research. In this study, we predict the spread of COVID-19 in the SADC region using confirmed daily cases from the 7^th^ of March 2020 to the 25^th^ of August 2021, yielding 535 observations. Since the data set is relatively large, training using the first 520 observations is done, then testing using the last 15 observations followed by a 14-forecast using the candidate models.

The single forecast models used in this study are the ARIMA models, TBATS model, GAM and the SGBM approaches. The GAM outperforms all the other models since has the lowest RMSE, MAPE and Theil's U statistic from these approaches. On testing the model's performance using plots, we discovered that the performance of the GAM was not outright. The GAM could not perform well from 18^th^ August, 2021, to the 20^th^ August, 2021. However, the SGBM and the TBATS models perform better in this interval. Thus, we decided to combine forecasts from the ARIMA, TBATS, GAM, and SGBM to ensure the final model's accuracy.

The forecasts from the single technique models are combined using the Quantile regression approaches, i.e., linear quantile regression averaging (LQR), Monotone Composite Quantile Regression Neural Network model (MCQRNN), PLAQR and the OPERA. The MCQRNN is a novel approach to nonlinear quantile regression modelling that: 1) simultaneously estimates multiple non-crossing, nonlinear conditional quantile functions, 2) allows for optional monotonicity, positivity and generalized additive model constraints, 3) can be adapted to estimate standard least-squares regression and non-crossing expectile regression functions^18^.

The combined forecasts models show an increased performance accuracy compared to the performance accuracies for the single forecast models. This is in congruency with findings from studies on combining time series, which purports that combining forecasts from different models effectively reduces the prediction errors and provides considerably increased accuracy^9^,^14^,^39^. Cross-validation results suggest that MCQRNN is more robust than all the other models (RMSE=0.00132, MAPE=0.00000614, Theil's U=0.000000278). Its Theil's U statistic is close to zero, indicating a perfect fit. The closer the Theil inequality coefficient is to 0, the smaller the difference between the predicted value and the real value will be, which indicates the better fitting degree of the prediction model^40^. A study on non-crossing nonlinear regression quantiles by MCQRNN on rainfall extremes also confirms the robustness of the MCQRNN approach compared to other baseline models^18^

We developed a quantile regression average model to perform a 14-day out of sample forecast. The model predicted a fairly decreasing trend from 22194 on the 26^th^ of August 2021 to 13189 on the 8^th^ of September 2021, on the number of confirmed cases in the SADC region. We further investigated the sharpness of the fitted models using the PINAWs from simple average and median at 95% level. The median showed a narrower prediction interval. Considering this as the first study conducted using the combined forecast approach to predict the spread of COVID-19, our findings significantly predict the pandemic. The approach allows for the timely forecasting of the spread of COVID-19, hence informing of the introduction of effective interventions in the SADC region.

## Conclusion

Forecasting plays an important role in decision making, particularly in this period where the COVID-19 pandemic is challenging the entire world. However, single forecasts techniques do not perform well in predicting the spread of COVID-19 in the SADC region. Combined forecasts models using quantile regression averaging increases accuracy in predicting COVID-19 cases. A prediction of a downward trend for the next 14 days in the COVID-19 cases is shown from the fitted combined forecast model. The findings present an insightful approach in monitoring the spread of COVID-19 in SADC region. The spread of COVID-19 in the SADC region can best be predicted using combined forecasts models, particularly the MCQRNN approach.

## Abbreviations

ACF: Autocorrelation function
ADF: Augmented Dickey-Fuller
AIC: Akaike information criteria
ARIMA: Autoregressive integrated moving average
COVID-19: Coronavirus Infectious Disease 2019
CVC: Cross-validation criterion
GAM: Generalised additive models
KPSS: Kwiatkovski-Phillips-Schmidt-Shin
LQRA: Linear quantile regression averaging
MAE: Mean absolute error
MAPE: Mean absolute percentage error
MCQRNN: Monotone composite QRNN
OPERA: Online prediction by ExpeRt aggregation
PACF: Partial ACF
PAQRA: Partial additive quantile regression averaging
PINAW: Prediction interval normalized average width
PIW: Prediction interval width
QRNN: Quantile regression neural network
RMSE: Root means square error
SADC: Southern African Development Community
SARIMA: Seasonal ARIMA
SGBM: Stochastic gradient boosting machine
TBATS: Trigonometric seasonality, Box-Cox transformation, ARIMA errors, Trend and Seasonal components
